# An often neglected area in crooked nose: middle turbinate pneumatization^☆^

**DOI:** 10.1016/j.bjorl.2016.06.006

**Published:** 2016-07-20

**Authors:** Fatih Özdoğan, Halil Erdem Özel, Erkan Esen, Erdem Altıparmak, Selahattin Genç, Adin Selçuk

**Affiliations:** Derince Research and Training Hospital, Department of Otolaryngology, Kocaeli, Turkey

**Keywords:** Turbinate, Crooked nose, Rhinoplasty, Nasal airway, Corneto, Nariz torto, Rinoplastia, Via respiratória nasal

## Abstract

**Introduction:**

Crooked or deviated nose is a deviation of the nose from the straight vertical position of the face. Extensive pneumatization of the middle turbinate, also called concha bullosa or bullous middle turbinate (BMT) is known to be one of the possible etiologic factors in nasal obstruction, recurrent sinusitis, and headache. There is no study concerning a link between BMT and crooked nose.

**Objective:**

To investigate the association between crooked nose and the presence of a BMT.

**Methods:**

A total of 199 patients who underwent open septorhinoplasty were retrospectively analyzed. Preoperative paranasal Computerized Tomography (CT) findings, preoperative photodocumentation, and anterior rhinoscopic examination findings were documented. Of the 199 patients, 169 were found to meet the criteria and were included in the study. CT scans were examined to note the presence of BMT, inferior turbinate hypertrophy, and septum deviation (SD). SDs and crooked noses were classified.

**Results:**

Ninety-four of 169 patients (56%) presented a crooked nose deformity and seventy-five of 169 patients (44%) presented a straight nose. While 49 (52%) crooked nose patients had a bulbous and extensive BMT, 20 patients with straight nose (26.6%) had a BMT. A statistically significant relationship was found between the presence of crooked nose and BMT, regardless of the side of the disease (*p* = 0.011).

**Conclusion:**

This study revealed a link between crooked nose and BMT.

## Resumo

**Introdução:**

O nariz torto ou o nariz com desvio é um nariz com um desvio da posição vertical reta da face. A pneumatização extensa da concha média também chamada de concha bolhosa ou concha média bolhosa (CMB) é conhecida por ser um dos possíveis fatores etiológicos da obstrução nasal, sinusite recorrente e cefaleia. Não há nenhum estudo relativo a uma associação entre CMB e nariz torto.

**Objetivo:**

Investigar a associação entre o nariz torto e a presença de CMB.

**Método:**

Foram analisados retrospectivamente 199 pacientes que se submeteram a septorrinoplastia aberta. Achados pré-operatórios paranasais à tomografia computadorizada (TC), fotodocumentação pré-operatória e exame rinoscópico anterior foram registrados. Dos 199 pacientes, observou-se que 169 atendiam aos critérios e foram incluídos no estudo. As TC foram examinadas para observar a presença de CMB, hipertrofia de conchas inferiores e desvio de septo (DS). Os DS e narizes tortos foram então classificados.

**Resultados:**

Dos 169 pacientes, 94 (56%) apresentavam uma deformidade de nariz e 75 (44%) apresentavam nariz reto. Enquanto 49 (52%) pacientes com nariz torto tinham CMB extensa, 20 pacientes com nariz reto (26,6%) tinham CMB. Uma relação estatisticamente significativa foi encontrada entre a presença de nariz torto e CMB, independentemente do lado da doença (*p* = 0,011).

**Conclusão:**

Este estudo revelou uma relação entre o nariz torto e CMB.

## Introduction

Crooked or deviated nose is a deviation of the nose from the straight vertical position of the face. In addition to esthetic deformity, functional problems due to obstruction of the airway can be seen in deviated nose. Crooked nose is associated with nasal obstruction, sinus headaches, and sinus infections. Most deviations involve several structures of the nose, and failure to correct all the abnormalities often results in disappointing outcomes. Therefore, it is essential to consider both the internal nasal structures such as the nasal valve and turbinates, and the external nasal frame.^1^, ^2^

Extensive pneumatization of the middle turbinate, also called concha bullosa or bullous middle turbinate (BMT), is known to be one of the possible etiologic factors in nasal obstruction, recurrent sinusitis, and headache.^3^, ^4^ Several studies have been focused on the relation between septum deviations (SD) that can be one of the main causes of nasal air airway obstruction and BMT.^5^, ^6^ However, to the best of our knowledge, there is no study concerning a link between BMT and crooked nose. The main objective of this study is to investigate the association between crooked nose and the presence of a BMT.

## Methods

A total of 199 patients who underwent open Septorhinoplasty (SRP) between May 2011 and February 2015 were retrospectively analyzed. This study was approved by the local ethics committee (KAEK 2014-232). Preoperative paranasal CT findings, preoperative photodocumentation, and anterior rhinoscopy examination findings were documented. The patients who underwent previous sinonasal surgery and had a major nasal trauma were excluded from the study (only patients with type 1 nasal injury history were included).^7^ A total of 169 patients with a nasal SD who had undergone SRP were included in the study. Paranasal sinus CT scans of 169 patients were studied retrospectively. Image sections were 1 mm thick.

**Figure 1:**
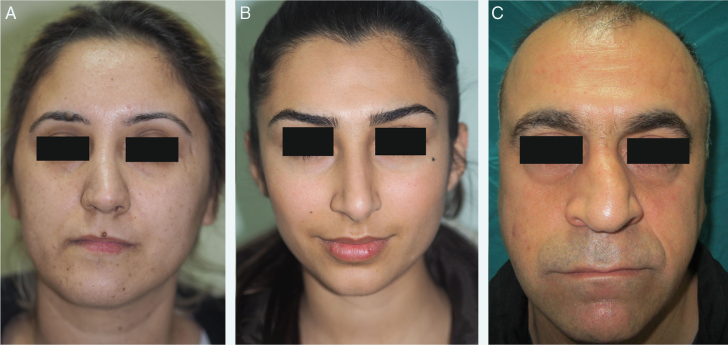
(A) Type I crooked nose (deviation of the lower two-thirds of nose). (B) Type II crooked nose (deviation of the whole nose in the same direction). (C) Type III crooked nose (deviation of the whole nose with a curved rhinion).

All patients underwent a detailed otolaryngologic examination and preoperative photography, consisting of frontal, basal, lateral, and oblique views. The criteria used for the classification of crooked nose were as follows (Fig. 1A–C)^8^:

Type I – Deviation of the lower two-thirds of nose;
Type II – Deviation of the whole nose in the same direction;
Type III – Deviation of the whole nose with a curved rhinion.

**Figure 2:**
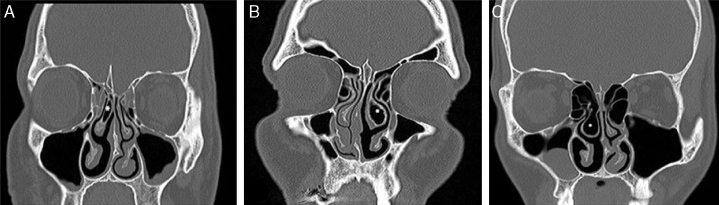
(A) Lamellar BMT (asterisk: pneumatized middle turbinate). (B) Bulbous BMT (asterisk: pneumatized middle turbinate). (C) Extensive BMT (asterisk: pneumatized middle turbinate).

CT scans were examined to note the presence of a BMT, inferior turbinate hypertrophy, and SD. BMTs were classified into three types according to the shape of the bullous change: lamellar BMT, bulbous BMT and extensive BMT. Only bulbous and extensive types of BMT were included in the study (Fig. 2A–C).^9^

The surgical procedures were performed under general anesthesia on all patients. All cases were operated upon using an open SRP approach via transcolumellar and marginal rim incision. The nasal suprastructure was exposed under the SMAS layer and skeletonization of the cartilaginous and bony framework. Patients with a bulbous and extensive BMT had also undergone partial BMT resection. The patients having inferior turbinate hypertrophy were performed radiofrequency thermal ablation.

Data were analyzed with Statistical Product and Service Solutions (SPSS), predictive analytics software (PASW), and Statistics 21 (SPSS Inc., Chicago, IL, USA). Chi-square tests were applied for the measurements; *p* < 0.05 was considered statistically significant.

## Results

Ninety-four of 169 patients (56%) presented a crooked nose deformity and seventy-five of 169 patients (44%) presented a straight nose. SD was present in all patients, which was confirmed by CT. Among patients with a crooked nose, 61 (65%) were males and 33 (35%) were females, with a mean age of 27.2 years (range 18–50 years). Among those with a straight nose, 42 patients (66%) were males and 33 (44%) were females, with a mean age of 29.8 years (range 18–54 years). The most common crooked nose type was type 3–47 patients (50%), followed by type 2–28 patients (29.7%), and type 1–19 patients (20.2%) (Table 1).

**Table 1:** Characteristics of all patients undergoing septorhinoplasty.

	Crooked nose	Straight nose
	*n* (%)	*n* (%)
*Gender*		
Male	61 (65)	42 (66)
Female	33 (35)	33 (44)
Total	94 (56)	75 (44)

*Age*	18–50 (27.2)	18–54 (29.8)

*Nasal deviation type*		
Type 1	19 (20.3)	–
Type 2	28 (29.7)	–
Type 3	47 (50)	–

*Nasal deviation side*		
Not-exist	–	75
Left	47 (50)	–
Right	47 (50)	–

*Septum deviation side*		
Left	55 (58.5)	44 (58.6)
Right	39 (41.4)	31 (41.3)

*Bullous middle turbinate type*		
Not-exist	30 (31.9)	42 (56)
Lamellar	15 (15.9)	13 (17.3)
Bulbous and extensive	49 (52.1)	20 (26.6)

*Bullous middle turbinate side*		
Not-exist	30 (31.9)	42 (56)
Left	29 (30.8)	4 (5.3)
Right	14 (14.8)	11 (14.6)
Bilateral	21 (22.3)	18 (24)

*Inferior turbinate hypertrophia*		
Not-exist	30 (31.9)	28 (37.3)
Left	18 (19.1)	7 (9.3)
Right	19 (20.2)	15 (15.9)
Bilateral	27 (28.7)	25 (33.3)

Bulbous and extensive BMT was detected in 49 out of the 94 patients who had a crooked nose (52%) and in 20 out of the 75 patients who had a straight nose (26.6%). A statistically significant relationship was found between the presence of crooked nose and BMT regardless of the side of the condition (*p* = 0.011). No significant correlation was observed between the type of nasal axis deviation and the presence of BMT. The distribution of patients according to the presence of nasal axis deviation and type of BMT is presented in Table 2.

**Table 2:** Distribution of patients according to presence of nasal axis deviation and type of bullous middle turbinate.

ND	BMT *n* (%)			*p*
*n* (%)	Not-exist	Lamellar	Bulbous and Extensive	
Not-exist	42 (56)	13 (17.3)	20 (26.6)	**0.011**
Exist	30 (31.9)	15 (15.9)	49 (52.1)	

Total	72 (42.6)	28 (16.6)	69 (40.8)	

ND, nasal deviation; BMT, bullous middle turbinate; *p*-values were determined using the Chi-squared test.

No significant correlation was observed between the side of nasal axis deviation and the side of BMT (*p* = 0.469). The distribution of patients according to the nasal axis deviation and side of BMT is presented in Table 3.

**Table 3:** Distribution of patients who had nasal axis deviation according to side of nasal axis deviation and side of bullous middle turbinate.

ND side	BMT side *n* (%)			*p*
*n* (%)	Right	Left	Total	
Not-exist	16 (17)	14 (14.9)	30 (31.9)	0.469
Right	8 (8.5)	6 (6.4)	14 (14.9)	
Left	11 (11.7)	18 (19.1)	29 (30.9)	
Bilateral	12 (12.8)	9 (9.6)	21 (22.3)	

Total	47 (50)	47 (50)	94 (100)	

ND, nasal deviation; BMT, bullous middle turbinate; *p*-values were determined using the Chi-squared test.

## Discussion

Extensive pneumatization of the middle turbinate, also called concha bullosa or bullous middle turbinate (BMT), is known to be one of the possible etiologic factors in nasal obstruction, recurrent sinusitis, and headache.^3^, ^4^ BMT is one of the most common anatomical variations that can be seen in the nasal cavity.^10^

Three types of middle turbinate pneumatization were described. In the first type, air cells were noted to pneumatize the vertical lamella of the turbinate. In the second type, air cells were noted to pneumatize the inferior or bulbous segment of the turbinate. In the third type, extensive pneumatization was observed in the lamellar and bulbous portion of the turbinate.^4^ The incidence of BMT mostly ranges from 14% to 53% in the literature^11^ Khojastepour et al.^12^ determined BMT variation in 189 (67.3%) of 281 rhinoplasty cases in their preoperative paranasal CT analysis. This proportion was 40% in all cases in our study.

The relationship between SD and BMT has been known for a long time. The incidence of coexistence of nasal SD and BMT is high. The relationship between BMT and nasal SD has been reported by Aktas et al.,^5^ Bhandary et al.,^13^ and Yigit et al.^6^ However, to the best of our knowledge, there is no study concerning a link between BMT and crooked nose. In our case series, we determined 49 crooked nose patients (52%) having a bulbous and extensive BMT and 20 patients with a straight nose (26.6%) and bulbous and extensive BMT. This association between BMT and crooked nose may be assumed as a condition that can occur after trauma by the deterioration of the nasal airway dynamics.

Externally nasal deviation always results in a deviation of the nasal septum. Saul et al.^14^ reported perpendicular plate deviation distorted to the reverse side of the deviation of the deviated nose in 79% of the patients. The most common SD type in our patients who had a crooked nose was posterior vertical deviation 23 patients (24.4%).

Preoperative evaluation and surgical management of the nasal airway in rhinoplasty patients are essential. According to a questionnaire applied to 671 members of the American Society of Plastic Surgeons by Afifi et al.,^15^ the question “in your preoperative exam middle turbinate routinely assess?” was answered as “yes” by 39.9% of the participants. In addition, 24.1% of the participants answered the question “If a patient presenting for an esthetic rhinoplasty has complaints of difficulty with breathing through the nose, would you perform the procedure?” as “do the esthetic rhinoplasty and a thorough septoplasty and turbinate resection.” The question of “how often do you address the middle turbinate during rhinoplasty?” was answered as never by 71% of the participants. These results suggest that the middle turbinate is often neglected in rhinoplasty. In our study, intranasal endoscopic examination was performed in all patients prior to performing SRP and preoperative CT. For patients having a BMT, partial resection of the turbinate was performed simultaneously as well.

Radiographic imaging is usually not a standard part of the workup in patients interested in rhinoplasty. However, it can be helpful in patients who may benefit from concurrent sinus surgery, those with rhinogenic migraines, or those with abnormal growth and development. Imaging can identify anatomic variations of the nasal bones and turbinates.^16^ We believe that preoperative CT is essential for patients with a crooked nose even if they do not have concomitant diseases (allergy, chronic sinusitis, and so on).

SRP, among elective facial surgeries, is one of the most frequent causes for litigation. Airway problems are the main concerns in several of these cases. Nasal obstruction is remarkably associated with a decrease in quality of life in these patients.^15^

Therefore, in patients scheduled for SRP (particularly with a crooked nose), the presence of BMT is one of the factors affecting the patient's nasal obstruction that must be detected as a preoperative intervention and managed.

## Conclusion

Sufficient information is unavailable in the literature on the incidence of the coexistence of crooked nose and BMT. Unfortunately, the presence of BMT in deviated nose has been ignored by most of the rhinoplasty surgeon. Our results indicate that BMT incidence is higher in patients with a crooked nose, for whom SRP has been planned, than in those with a straight nose. Therefore, in these patients, a detailed preoperative examination should be performed, the presence of BMT must be detected and shown with a paranasal CT scan, and BMT intervention should be conducted simultaneously with SRP. The most important limitation of this study is that postoperative airway and nasal dryness were not evaluated. Postoperative functional results can be evaluated by prospective studies.

## Conflicts of interest

The authors declare no conflicts of interest.
